# Policing and access to harm reduction services among young people who use drugs and young Indigenous people who use drugs before and after the pilot implementation of decriminalization of personal possession

**DOI:** 10.1016/j.drugpo.2025.105068

**Published:** 2025-11-14

**Authors:** Erica McAdam, M-J Milloy, Eric C. Sayre, Carmen Verdicchio, Kali-olt Sedgemore, Helena May, Samantha Pranteau, Dyami Corriveau, Drew Friesen, Mathew Fleury, Danya Fast, Kora DeBeck

**Affiliations:** a British Columbia Centre on Substance Use, Vancouver, BC, Canada; b Interdisciplinary Studies Graduate Program, University of British Columbia, Vancouver, BC, Canada; c Division of Social Medicine, Department of Medicine, University of British Columbia, Vancouver, BC, Canada; d ARYS Community Research Associates, BC Centre on Substance Use, Vancouver, BC, Canada; e Indigenous Collaborators Circle, BC Centre on Substance Use, Vancouver, BC, Canada; f British Columbia Children’s Hospital Research Institute, Vancouver, BC, Canada; g Foundry British Columbia, Vancouver, BC, Canada; h School of Public Policy, Simon Fraser University, Vancouver, BC, Canada

**Keywords:** Decriminalization, Harm reduction, Young people who use drugs, Criminalization, Policing, Substance use

## Abstract

**Background:**

On January 31, 2023, the province of British Columbia, Canada, implemented a pilot decriminalization of personal possession of certain drugs. This study investigated temporal trends in policing-related barriers to accessing harm reduction services among young people who use drugs (PWUD) in Vancouver before and after decriminalization.

**Methods:**

Data from 2021–2024 were collected from an open prospective cohort of street-involved young PWUD. Logistic regression with generalized estimating equations (GEE) assessed trends in self-reported policing-related barriers to harm reduction services before and after decriminalization, with calendar time and decriminalization (plus their interaction) as primary explanatory variables. A sub-analysis was conducted among participants who identified as being of Indigenous ancestry.

**Results:**

Among 319 participants, the median baseline age was 28.0 years, and 83 (26 %) reported policing-related barriers to harm reduction services at some point during the study period. In multivariable GEE analysis, an increasing trend in reporting police barriers was observed before decriminalization (adjusted per-year odds ratio [AOR]=2.41; 95 % confidence interval [CI]: 1.29–4.51). At the implementation of decriminalization, a significant level drop of 65 % was observed (AOR=0.35; 95 % CI: 0.15–0.82), with no trend observed post-decriminalization (AOR=1.12; 95 % CI: 0.48–2.58 per year). In sub-analysis among Indigenous participants, a significant decreasing trend of 72 % per year in reported policing-related barriers was observed in the post-decriminalization period (AOR = 0.28; 95 % CI: 0.08–0.97).

**Implications:**

Among young PWUD, we observed relative reductions in experiencing policing-related barriers to harm reduction services after the pilot implementation of decriminalization, and this benefit extended to young Indigenous PWUD.

## Introduction

Decriminalization of personal possession of unregulated drugs is a policy approach used by a few jurisdictions globally as an attempt to reduce the harms stemming from drug criminalization (Global Commission on Drug Policy, 2016, 2021; Henry, 2019; Hughes & Stevens, 2010). It is well-documented that drug criminalization has significant adverse consequences at both the individual and societal level (Csete et al., 2016; DeBeck et al., 2017; Global Commission on Drug Policy, 2013; Jensen et al., 2004; Khenti, 2014; Virani & Haines-Saah, 2020; Wodak, 2014). Specifically, policing practices targeting people who use drugs contributes to driving substance use into concealed, higher-risk environments, increasing overdose risk, and creating barriers to accessing critical health and harm reduction services (BC Ministry of Mental Health and Addictions, 2021; Csete et al., 2016; DeBeck et al., 2017; Virani & Haines-Saah, 2020). In response, multiple jurisdictions have implemented decriminalization as a step away from punitive drug policies, most notably Portugal. Evidence from these contexts suggests that decriminalization can reduce drug-related stigma, improve access to healthcare services, and decrease interactions with law enforcement (Benfer et al., 2018; Eastwood et al., 2016; Global Commission on Drug Policy, 2016; Henry, 2019; Hughes & Stevens, 2007; Hughes & Stevens, 2010; Unlu et al., 2020). Nevertheless, the effectiveness and outcomes of decriminalization depend on how policies are designed and implemented, in addition to the overall policy/political environment in which they occur (Scheim et al., 2020).

Canada and the United States are experiencing a catastrophic toxic drug crisis with unprecedented overdose-related mortality (Ahmad et al., 2024; Government of Canada, 2024; Humphreys et al., 2022; Spencer et al., 2024). In Canada, the opioid toxicity mortality rate almost tripled from 7.8 per 100,000 population in 2016 to 21.5 per 100, 000 population in 2023 (Government of Canada, 2024). The province of British Columbia (BC) has among the highest rates of toxic drug deaths, reaching 46.8 per 100,000 population in 2023 (BC Coroners Service, 2024). In response, the Canadian federal and some provincial governments have implemented harm reduction and public health strategies aimed at reducing overdose deaths, including in BC programs like the distribution of free naloxone kits and the expansion of overdose prevention sites (BC Ministry of Mental Health and Addictions, 2018, 2021).

In the context of the ongoing crisis, the BC government applied to the federal government for a specific exemption to Canada’s laws criminalizing possession of unregulated drugs. Specifically, the Government of BC was granted a Section 56(1) exemption from the *Controlled Drugs and Substances Act* to decriminalize personal possession of up to 2.5 grams of opioids, cocaine/crack, crystal methamphetamine, and MDMA (BC Ministry of Mental Health and Addictions, 2021; Health Canada, 2024b). Under BC’s decriminalization framework, police are prohibited from sanctioning or seizing drugs from individuals found to be in possession for personal use (BC Ministry of Mental Health and Addictions, 2021). Although the original exemption request decriminalized the possession of drugs in public places, with a number of limited exemptions (e.g., school grounds, airports, and motor vehicles), this scope was narrowed in May 2024 following political and public backlash (Health Canada, 2024b). The amended exemption significantly revised decriminalization to only apply to a limited number of private settings, including harm reduction and addiction treatment sites, locations where unhoused individuals are legally sheltering, and private residences (BC Ministry of Mental Health and Addictions, 2024b; Health Canada, 2024b). While police have been encouraged to exercise discretion and avoid enforcement in situations that do not pose a public safety risk, the revised exemption significantly narrows the scope of decriminalization (BC Ministry of Mental Health and Addictions, 2024b; Health Canada, 2024a).

Prior to the implementation of decriminalization, BC reported the highest rate of personal possession offences in Canada, surpassed only by the Northwest Territories in 2022. In 2021, BC recorded 157.8 offences of personal possession per 100,000 population—nearly triple the national average (excluding BC) of 54.8 per 100,000 (Statistics Canada, 2024). In the year following the implementation of decriminalization, there has been a substantial decline in personal possession offences—a 66 % decrease in the number of offences compared to the previous year (BC Ministry of Mental Health and Addictions, 2024a). In the first year of decriminalization, the rate of offences dropped to 44.6 per 100,000 population, aligning with the national average of 45.1 per 100,000 population (Statistics Canada, 2024). However, following the reversal of decriminalization in public places in May 2024, the BC Ministry of Health reports that the monthly average number of possession offences has increased to approximately 79 % of pre-decriminalization levels (BC Ministry of Health, 2025).

According to the Government of BC, the overarching objective of the decriminalization pilot project was to “reduce stigma and fear of criminal prosecution that prevents people from reaching out for help, including medical assistance” (BC Ministry of Mental Health and Addictions, 2021; Government of British Columbia, 2024). The policy also aimed to reduce barriers to accessing health and harm reduction services for people who use drugs (BC Ministry of Mental Health and Addictions, 2021). Prior research has documented that police presence near harm reduction and treatment services (including supervised consumption, needle distribution, overdose prevention, and drug checking sites) often discourages people who use drugs from accessing these services due to fears of criminal prosecution/sanction, harassment, or drug seizure (Bardwell et al., 2019; Beletsky et al., 2014; Collins et al., 2019; DeBeck et al., 2017; Goldenberg et al., 2020; Grace Rose et al., 2023; Krug et al., 2015; Petrar et al., 2007; Watson et al., 2021; Weisenthal et al., 2022). These concerns are particularly acute for young people who use drugs, who experience disproportionate targeting by police compared to adults who use drugs (Card et al., 2021; Greer et al., 2021; Greer, Selfridge, et al., 2022; Greer et al., 2020; Greer et al., 2018; Ti et al., 2013). Indigenous youth who use drugs face heightened criminalization-related harms due to both historic and ongoing colonial and systemic police violence (Card et al., 2021; Pan et al., 2013). For example, research from our setting has documented how young Indigenous youth experience increased rates of contact with the criminal justice system compared to non-Indigenous youth even after controlling for drug use patterns and other risk behaviours (Barker et al., 2015).

While the link between policing and barriers to harm reduction services is well-described, there remains a paucity of peer-reviewed research investigating the relationship between decriminalization and barriers to harm reduction services. Government reports monitoring the impacts of decriminalization have noted increased use of overdose prevention, supervised consumption, and drug-checking sites since the pilot began (BC Ministry of Mental Health and Addictions, 2024c). However, this aggregated data does not account for potential confounding or external factors that may have influenced service utilization, such as the expansion of these sites or increases in operating hours. Furthermore, survey data indicates that 14 % of people who use drugs in BC continue to be hesitant to access harm reduction services due to fear of police confiscation of substances (BC Ministry of Mental Health and Addictions, 2024c). It remains unclear what, if any, impact decriminalization in BC had on policing-related barriers to accessing harm reduction services prior to the revision of the exemption in May 2024. To address this gap, we sought to examine temporal trends in self-reported policing-related barriers to harm reduction services (including supervised consumption, overdose prevention, and drug checking sites) among young people who use drugs in Vancouver, BC, before, upon, and after the implementation of decriminalization of personal possession (prior to the policy change in May 2024 limiting decriminalization to select private settings). We also sought to investigate if there were any associations between decriminalization and policing-related barriers to harm reduction sites among young Indigenous people who use drugs, given their disproportionate exposure to police violence and criminalization. Understanding the relationship between decriminalization and self-reported policing-related barriers to harm reduction services is a central aspect of evaluating the policy’s effectiveness in reducing criminalization-related harms and informing refinements to maximize public health benefits, particularly for marginalized populations most affected by drug enforcement.

## Methods

We used data from the At-Risk Youth Study (ARYS), an ongoing prospective cohort study of street-involved young people who use drugs in Vancouver, Canada, which began in 2005. The study design and methodology have been previously described in detail (Wood et al., 2006). Briefly, to be eligible to enroll in ARYS, individuals must be between the ages of 14 to 26 years at baseline, live in the Greater Vancouver Regional District, have used unregulated drugs (other than or in addition to cannabis, alcohol, and tobacco) in the past month, and be street-involved, defined as being without stable housing or utilizing services for youth who are experiencing homelessness within the past month. All ARYS participants provide written informed consent to participate in the study. Participants do not “age out” of ARYS; rather, they are invited to continue participating in the study and if younger age is a relevant consideration, data are restricted at the analysis stage. Data are collected through interviewer-administered questionnaires at baseline and every six months thereafter, capturing information on demographics, substance use patterns and associated risks, income-generating activities, interactions with the police, engagement with health and social services, among others. Before October 31, 2022, participants received a $40 CAD honorarium for each study visit. After November 1, 2022, the honorarium was increased to $50 CAD for each study visit. In response to the COVID-19 pandemic in BC, study interviews were held remotely via telephone or videoconferencing between March 2020 and February 2022 for participants already enrolled in ARYS. In-person study interviews recommenced in March of 2022. Data collection procedures during the pandemic have been described in detail and published elsewhere (McAdam et al., 2022). The Providence Health Care, University of British Columbia Clinical, and Simon Fraser University Research Ethics Boards have approved the ARYS cohort.

The decision to examine the relationship between decriminalization and policing-related barriers specifically among young Indigenous participants was guided by discussions with our team’s Indigenous Collaborators Circle. The Circle comprises six individuals who identify as Indigenous (i.e., First Nations, Métis, and/or Inuit) with lived and/or living experience of substance use, who meet regularly to guide research activities of ARYS and the cohorts with which ARYS is harmonized, as it pertains to Indigenous participants and their data. In addition to offering lived expertise and cultural guidance, the Circle plays a key role in advancing Indigenous data governance by informing ethical approaches to data collection, analysis, storage, and use. Their involvement ensures that research is conducted in a way that respects Indigenous self-determination, upholds principles of OCAP^®^ (Ownership, Control, Access, and Possession), and centers community priorities throughout the research process. The Circle contributed to the planning and interpretation of this analysis.

Participants who completed a study interview between June 2021 and May 2024, answered the main outcome question (policing-related barriers to harm reduction services), and reported recent unregulated drug use in the same follow-up period that they answered the main outcome question, were included in this study. Data for the analyses were restricted to observations where a participant was 35 years of age or younger at each response. To assess the level and trends in policing-related barriers to harm reduction services, we defined our primary outcome of interest as having experienced policing-related barriers to accessing harm reduction services in the last six months (yes vs. no), ascertained through the question: “In the last six months, has police presence near a supervised consumption, overdose prevention or drug checking site prevented you from using the facility?” The primary explanatory variables of interest were calendar time in years (representing the pre-decriminalization trend), decriminalization (representing immediate level change upon the implementation of decriminalization, defined as an interview on or after January 31, 2023; yes vs. no), and a time by decriminalization interaction term (representing the trend post-decriminalization).

We selected a range of secondary explanatory variables we hypothesized might confound the relationship between policing-related barriers and decriminalization. All variables referred to the six month period before the interview, except for gender and race/ancestry, unless otherwise specified. Demographic factors included: self-identified gender (woman, transgender, Two-Spirit, and those who preferred to self-describe vs. man); age (per year older); strongest self-identified race/ancestry (Black, Indigenous, or Person of Colour vs. white); born in Canada (yes vs. no); and self-identified 2SLGBT identity (2SLGBT and those who self-described their identity vs. male, female, straight, heterosexual). Socioeconomic factors included: educational attainment (< high school diploma vs. ≥ high school diploma); experiencing homelessness (yes vs. no); unstable housing at the time of interview, defined as living in a single-room occupancy hotel, shelter, treatment facility, jail, and other unstable housing situations (yes vs. no); Downtown Eastside (DTES) residence, a neighbourhood in Vancouver with a well-characterized open drug scene (Wood et al., 2004) (yes vs. no); regular employment, defined as having a regular job, temporary work, or self-employed (yes vs. no); engagement in sex work, defined as receiving money, gifts, food, shelter, clothes, or drugs for sex (yes vs. no); involvement in drug dealing, defined as receiving money in exchange for drugs (yes vs. no); engaging in illegal income generating activities, including theft, robbing, fraud, other illegal actives, excluding sex work and drug dealing (yes vs. no); and recent incarceration (yes vs. no). Substance use related factors included: at least weekly unregulated opioid use, defined as reporting any use of fentanyl, down unspecified, heroin, and/or other illicit opioids (yes vs. no); at least weekly cocaine use (yes vs. no); at least weekly crack cocaine use (yes vs. no); at least weekly crystal methamphetamine use (yes vs. no); binge drug use, defined as using drugs more often than usual (yes vs. no); inject drugs alone (yes vs. no); and public injection (yes vs. no). Health service engagement factors included: opioid agonist therapy (OAT), including methadone, buprenorphine/naloxone, long-acting buprenorphine (e.g., Sublocade), methadose, methadol-D, slow release oral morphine (e.g., Kadian), long acting oral morphine, injectable hydromorphone, injectable diacetylmorphine, and other injectable opioids (yes vs. no); any drug or alcohol treatment, defined as having engaged with any drug or alcohol treatment programme, including detox/withdrawal management, a recovery house, a treatment centre, a counsellor, Narcotics Anonymous/Cocaine Anonymous/Alcoholics/Anonymous/Self-Management and Recovery Training (SMART), out-patient treatment, or drug treatment court (excluding OAT) (yes vs. no); and experienced barriers to accessing addiction treatment (yes vs. no). Health status and outcome factors included: non-fatal overdose, defined as an acute negative reaction following drug use (yes vs. no); any injection drug use (yes vs. no); moderate or severe depression in the past seven days (yes vs. no); and moderate or severe anxiety in the past seven days (yes vs. no), assessed by their respective Patient-Reported Outcomes Measurement Information System (PROMIS) short form measures (Pilkonis et al., 2011). PROMIS short form scales for depression range from 8 to 40 and for anxiety range from 7 to 35, with higher scores indicating greater severity (American Psychiatric Association, 2013). Total raw scores were converted into T-scores, and we dichotomized depression and anxiety variables at T-scores ≥60.0, indicating moderate/severe, and scores ≤59.9 interpreted as none/mild (American Psychiatric Association, 2013).

We examined baseline characteristics of study participants using combined data from: i) the first study visit from each participant who did not ever report policing-related barriers during the study period; and ii) the first survey response with a positive report of policing-related barriers for those who did report policing-related barriers during the study period, among the entire sample and a subsample of ARYS participants who identified as being of Indigenous ancestry. Baseline characteristics were stratified by whether participants had experienced policing-related barriers to accessing harm reduction sites. Exact chi-square tests were used to compare categorical variables, and Kruskal-Wallis tests were used to compare continuous variables. We examined policing-related barriers to harm reduction sites during study follow-up using logistic regression with generalized estimating equations (GEE), with an exchangeable correlation structure for the analysis of correlated data. Bivariate logistic models were fit in the overall sample. Next, a multivariable model was selected via confounder selection as follows. Beginning with a fully adjusted model that included all covariates significant at *p* < 0.10 in the bivariate analyses, we employed a manual stepwise approach to drop the potential confounder with the smallest maximum relative impact on the odds ratios of the three decriminalization-related explanatory variables compared to the odds ratios in the fully adjusted model. Next, we removed the potential confounder with the smallest maximum relative impact on the odds ratios of the decriminalization explanatory variables compared to the odds ratios in the reduced model. This step was repeated until the smallest maximum relative change, resulting from dropping any remaining potential confounders, was ≥5 % compared to the previously reduced model odds ratios, at which point no additional variables were dropped.

In order to explore whether there was a relationship between decriminalization and policing-related barriers to harm reduction sites among young Indigenous people who use drugs, we conducted a sub-analysis among participants who identify as Indigenous, repeating the methods described above.

All statistical analyses were performed using SAS version 9.4 (SAS Institute Inc., Cary, NC, USA). All p-values are two-sided and considered statistically significant at p≤0.05.

## Results

Between June 2021 and May 2024, 319 participants were seen for a study visit and were eligible for inclusion in this analysis. The median age of the sample was 28.0 years (interquartile range [IQR]: 24.9–30.4) at the first study visit when police barriers were reported, and for participants who did not report police barriers, age was derived from their first study visit during the study period. Among this sample, 100 (31.3 %) participants self-identified as a woman, 6 (1.9 %) were transgender, 9 (2.8 %) identified as Two-Spirit, and 12 (3.8 %) self-described their gender identity; 138 (43.3 %) individuals identified as being of Indigenous ancestry (regardless of strongest identity), 31 (9.7 %) identified as a Person of Colour, 13 (4.1 %) identified as Black, and 165 (51.7 %) identified as white. The 319 participants included in this analysis contributed 937 observations, with a median of 3 study visits per participant (interquartile range [IQR]: 1–4), and a median follow-up time of 12.9 months (IQR: 0.0–26.9).

**Fig. 1.**
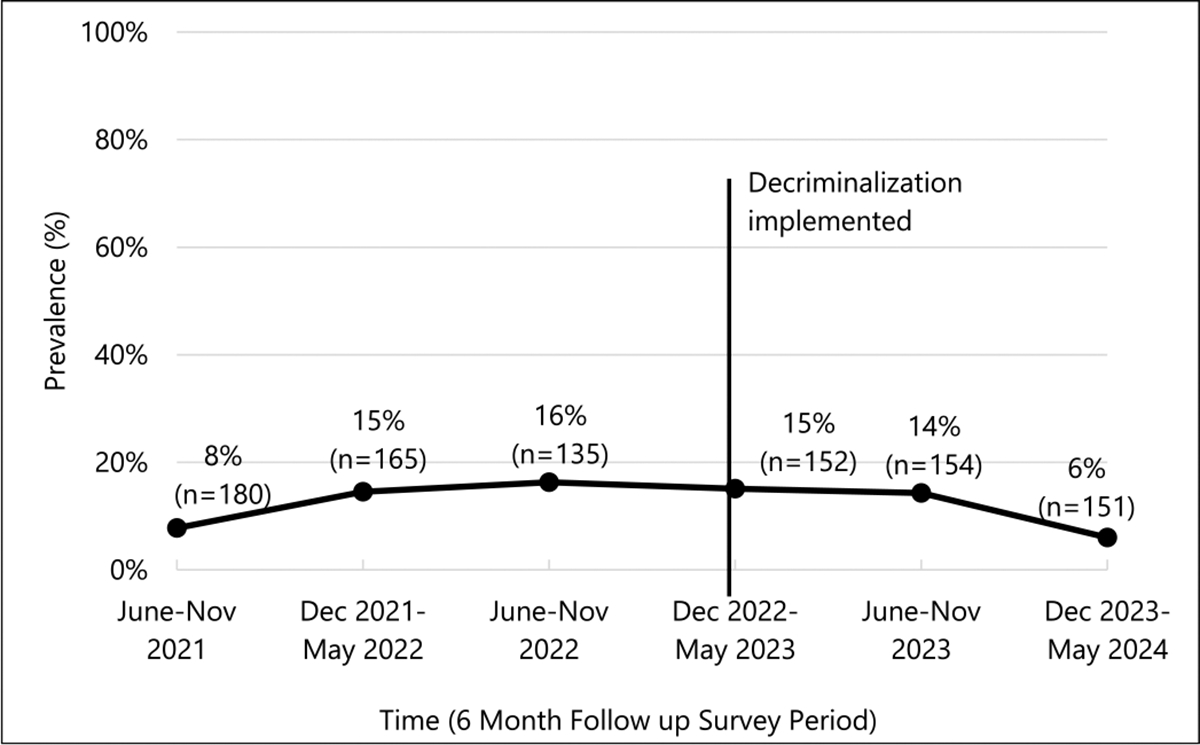
Prevalence rates of experiencing policing-related barriers to accessing harm reduction services among street-involved young people who use drugs in Vancouver, Canada, between June 2021-May 2024, by follow-up survey (*n* = 937 observations).

Overall, 83 (26.0 %) participants reported experiencing policing-related barriers to accessing harm reduction services at some point during the study period. Fig. 1 depicts the prevalence of reports of experiencing police barriers by survey follow-up period. In the pre-decriminalization period (June 2021-January 2023; *n* = 520 observations), 67 (12.9 %) study visits included a report of experiencing police barriers, and in the post-decriminalization period (February 2023-May 2024; *n* = 417), 47 (11.3 %) study visits included a report of police barriers. Table 1 provides characteristics of study participants stratified by experiencing policing-related barriers to harm reduction sites (for participants who did not report police barriers, descriptives are taken at the first study visit during the study period, and for participants who did experience police barriers, descriptives are taken from the first study visit of a positive report).

The results of the bivariable and multivariable GEE analyses among all included participants are shown in Table 2. In the multivariable GEE model, which adjusted for a range of secondary factors, pre-decriminalization there was a significant increasing trend in reports of experiences of policing-related barriers to harm reduction sites over time (per-year adjusted odds ratio [AOR]: 2.41; 95 % Confidence Interval [CI]: 1.29–4.51); however, upon the implementation of decriminalization, there was a significant level drop of reports of policing-related barriers to harm reduction services (AOR: 0.35; 95 % CI: 0.15–0.82). In the post-decriminalization period, no significant trend in reporting policing-related barriers to harm reduction was observed (per-year AOR: 1.12; 95 % CI: 0.48–2.58).

In a sub-analysis among ARYS participants who identified as having Indigenous ancestry, 138 individuals were eligible for inclusion. The median age of the sample was 27.1 years (IQR: 24.8–29.8) at the first study visit when police barriers were reported, and for participants who did not report police barriers, age was derived from the first study visit during the study period. 44 (31.9 %) participants self-identified as a woman, 1 (0.7 %) participant was transgender, 8 (5.8 %) identified as Two-Spirit, and 5 (3.6 %) self-described their gender identity. The 138 participants included in the sub-analysis contributed 386 observations, with a median of 2 study visits per participant (interquartile range [IQR]: 1–4), and a median follow-up time of 10.6 months (IQR: 0–25.3).

**Fig. 2.**
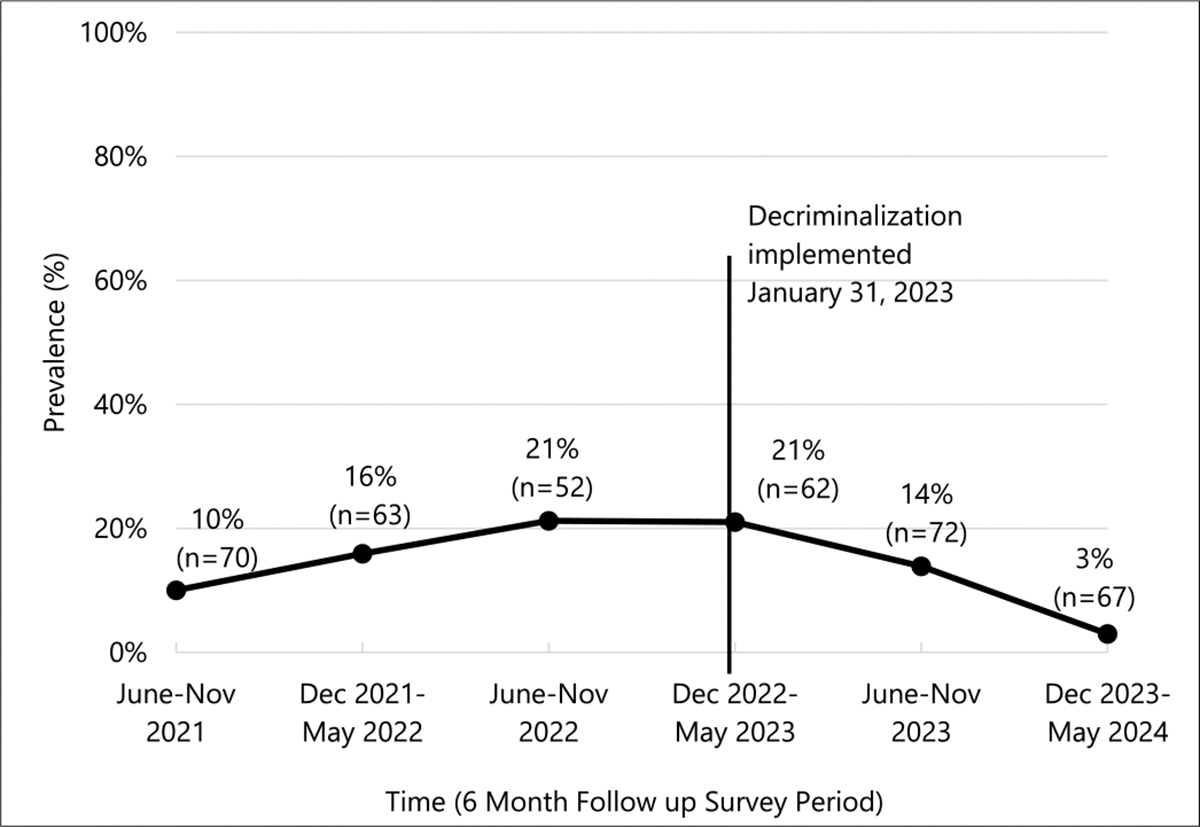
Prevalence rates of experiencing policing-related barriers to accessing harm reduction services among street-involved young Indigenous people who use drugs in Vancouver, Canada, between June 2021-May 2024, by follow up survey (*n* = 386 observations).

Overall, 39 (28.3 %) Indigenous participants reported experiencing policing-related barriers to accessing harm reduction sites at some point during the study period. Fig. 2 depicts the prevalence of reports of experiencing police barriers by survey follow-up period. In the pre-decriminalization period (June 2021-January 2023; *n* = 199 observations), 32 (16.1 %) study visits included a report of experiencing police barriers. In the post-decriminalization period (February 2023-May 2024; *n* = 187), 21 (11.2 %) study visits included a report of police barriers. Table 3 provides characteristics of Indigenous participants stratified by experiencing policing-related barriers to harm reduction sites.

The results of the bivariable and multivariable GEE sub-analyses among Indigenous participants are presented in Table 4. In the multivariable GEE model, which adjusted for a range of secondary factors, there was a significant increasing trend in reports of experiencing policing-related barriers to harm reduction sites over time before decriminalization (per-year AOR: 2.44; 95 % CI: 1.11–5.37). At the implementation of decriminalization, there was no significant level change in policing-related barriers observed (AOR: 0.66; 95 % CI: 0.22–1.98); however, in the post-decriminalization period, there was a significant declining trend in policing-related barriers (per-year AOR: 0.28; 95 % CI: 0.08–0.97).

## Discussion

In this study of young people who use drugs in Vancouver, Canada, more than one-quarter of the participants reported experiencing policing-related barriers to accessing harm reduction services between June 2021 and May 2024. In multivariable GEE analysis, which adjusted for a range of individual, social, structural, and environmental factors, before the implementation of decriminalization, we observed a significant increasing trend, an average of 141 % per year, in the odds of young people who use drugs reporting that they experienced policing-related barriers to accessing harm reduction sites. At the implementation of decriminalization, this increasing trend reversed, and we observed a 65 % level drop in the odds of reporting policing-related barriers to harm reduction services. A similar pattern was found in the sub-analysis among Indigenous participants: pre-decriminalization, there was a significant increasing trend, an average of 144 % per year, in the odds of reporting policing-related barriers to harm reduction services. At the implementation of decriminalization, no significant level change was observed; however, post-decriminalization, a significant declining trend was observed, with an average decline of 72 % per year in reported policing-related barriers to harm reduction.

One potential interpretation of the observed decline in experiencing policing-related barriers is that there was a reduction in police around harm reduction sites during decriminalization. Provincial government documents state that an objective of decriminalization was to reduce police resources spent on enforcement of personal possession, enabling police to shift to focus on more serious crime; however, these documents also acknowledge that police will likely still be interacting regularly with people who use drugs (BC Ministry of Mental Health and Addictions, 2021). Police are heavily influenced by the actions of criminal prosecutors, who have been directed in the Canadian context as of August 2020 not to pursue simple charges for personal possession (Greer, Zakimi, et al., 2022; Public Prosecution Service of Canada, 2020). The guideline directs prosecutors to focus only on the “most serious manifestations” of personal possession, specifically where aggravating public safety factors are present (e.g., conduct posing a risk to others, particularly children; association with other criminal conduct; possession that breaches the rules within regulated setting, such as correctional facilities) (Public Prosecution Service of Canada, 2020). In all other cases, prosecutors are directed to pursue diversion measures (Public Prosecution Service of Canada, 2020). This directive has effectively resulted in a form of *de facto* decriminalization across Canada, signaling to police that simple possession without aggravating factors is unlikely to be prosecuted. Consistent with this interpretation, qualitative research with police officers in BC prior to decriminalization documented a sentiment among police officers that charging for personal possession was a waste of time, given that prosecutors often do not pursue these cases (Greer, Zakimi, et al., 2022). There remains, however, broad police discretion even within the context of decriminalization of personal possession, meaning that individual officers may still be present near harm reduction sites, even if not actively enforcing personal possession laws. As described in the qualitative study by Greer *et al*., police often discuss shifting their efforts from enforcing personal possession to trafficking, which often results in focusing on more visible, street-based trafficking (Greer, Zakimi, et al., 2022). Additionally, qualitative research with people who use drugs in BC during decriminalization found that some participants from Vancouver reported continued or, in some cases, increased police presence near health and harm reduction sites, which they described as a barrier to service access (Wood, 2022). Our findings are consistent with these qualitative studies: While the overall likelihood of encountering policing-related barriers decreased, such barriers did not disappear entirely.

Another possible explanation of our findings is that police presence at harm reduction sites remained constant but was perceived as less threatening due to the protections afforded by the decriminalization of personal possession framework. This interpretation may be less likely given the long history of police violence and injustice towards young people who use drugs—particularly Indigenous youth—in Vancouver and British Columbia (Barker et al., 2015; Card et al., 2021; Greer et al., 2021; Greer, Selfridge, et al., 2022; Greer et al., 2018; Greer et al., 2024; Hayashi et al., 2023; O’Grady et al., 2011; Pan et al., 2013; Ti et al., 2013; Werb et al., 2008; Wood, 2022). Qualitative research post-decriminalization has documented persistent fears of police among structurally-marginalized youth (Wood, 2022). This research also found that youth who use drugs who were more “socially integrated” (i.e., less socioeconomically marginalized; based on degree of housing and employment) reported fewer and less harmful police interactions (Wood, 2022). Similarly, data from the BC Centre for Disease Control Harm Reduction Survey found that post-decriminalization, 14 % of people who use drugs indicated hesitance to access services as a result of fears of police confiscation of drugs (Fraser et al., 2024). Awareness of decriminalization may have also influenced participants’ perceptions of police presence acting as a barrier. A study conducted by Greer et al. evaluating awareness of decriminalization among people who use drugs accessing harm reduction services documented that 75 % of participants sampled in Vancouver were aware of decriminalization (Greer et al., 2024). However, knowledge of the specific details of decriminalization was mixed, with many participants conflating decriminalization with other drug policies (Greer et al., 2024). Together, these findings underscore that perceptions of police as a barrier are shaped not only by formal policy changes but also by individuals’ awareness of decriminalization, prior experiences with police, and the intersecting social positions (which may interact to make an individual more vulnerable to negative police interactions). It is likely that both mechanisms—a potential reduction in police presence and shifting perceptions among some subgroups who are less marginalized—contributed to the observed decrease in reported policing-related barriers to harm reduction services.

To our knowledge, our study is the first to examine temporal trends in policing-related barriers to harm reduction services before and after the implementation of decriminalization in BC, Canada. While literature from other jurisdictions—most notably Portugal—has shown improved access to services following decriminalization, these improvements are generally attributed to reductions in stigma and increased service investment, rather than changes in police practices specifically (Benfer et al., 2018; Hughes & Stevens, 2007; Hughes & Stevens, 2010; Unlu et al., 2020). In Portugal, increases in access to harm reduction and treatment services observed after the implementation of decriminalization have been attributed to both decriminalization and the significant expansion of harm reduction services that followed (Eastwood et al., 2016; Greenwald, 2009; Hughes & Stevens, 2007; Mendes et al., 2019).

Overall, our findings indicate that the implementation of decriminalization of personal possession was positively associated with a key policy objective to reduce reported barriers to health and harm reduction services. This is particularly meaningful given the substantial evidence documenting the harms associated with police presence near harm reduction and treatment sites (Bardwell et al., 2019; Beletsky et al., 2014; Collins et al., 2019; DeBeck et al., 2017; Goldenberg et al., 2020; Grace Rose et al., 2023; Krug et al., 2015; Petrar et al., 2007; Watson et al., 2021; Weisenthal et al., 2022). Importantly, we observed that the post-decriminalization time period was also associated with a significant declining trend in reported police barriers to harm reduction among young Indigenous people who use drugs, which is noteworthy given the greater harms experienced by this group as a result of historic and ongoing criminalization and systemic racist police violence (Barker et al., 2015; Card et al., 2021; Pan et al., 2013). There is a lack of disaggregated data on the demographic characteristics of individuals charged with personal possession, making it difficult to assess whether decriminalization has reduced racial disparities in drug law enforcement. However, national incarceration statistics demonstrate sustained inequities in criminal legal system involvement—in 2023, Indigenous peoples represented approximately 5 % of the population yet account for 29 % of all federally sentenced individuals, and Indigenous women comprise 50 % of all federally incarcerated women (Department of Justice Canada, 2024; Public Safety Canada, 2023). While the declining trend in policing-related barriers among Indigenous participants in our study is encouraging, these findings must be interpreted within the broader context of systematic over policing and the ongoing criminalization of Indigenous peoples.

It is important to note that BC’s decriminalization framework was substantially revised in May 2024, effectively re-criminalizing personal possession in public places (BC Ministry of Mental Health and Addictions, 2024b; Health Canada, 2024b). This policy reversal is concerning, particularly in light of early indicators of success in reducing interactions with the criminal justice system among people who use drugs (BC Ministry of Mental Health and Addictions, 2024c; Wood, 2022). It is unclear whether policing-related barriers to accessing harm reduction services will continue to decline, stay the same, or increase, following the reversal of decriminalization in BC. Future research is warranted to assess the impact of these changes.

The findings from our study should be interpreted in the context of several limitations. Participants in the ARYS cohort were not recruited randomly, as it is not possible to generate a random sample of hard-to-reach populations, such as street-involved youth; therefore, our findings cannot be generalized to other young people who use drugs in Vancouver, Canada, or to other settings. However, the ARYS cohort is believed to be reflective of structurally marginalized young people who use drugs. For example, ARYS is aligned with other study samples of young people who use drugs, such as the ‘RAPIDS’ study in Rhode Island (rates of active homelessness are high: 20.6 % in ARYS and 18 % in RAPIDS; regular opioid use: 40.9 % in ARYS and 37 % in RAPIDS) (Krieger et al., 2018). We controlled for several relevant secondary factors in our analysis; however, we cannot rule out the possibility of residual confounding. Reports of experiences of policing-related barriers and other substance use factors were ascertained via self-report, which may lead to social desirability or recall bias. However, previous research has demonstrated that self-reported drug use behaviours among people who use drugs are valid and reliable (Bharat et al., 2023; Darke, 1998). Our primary outcome measure was based on a dichotomous measure assessing whether participants perceived police presence as a barrier to accessing harm reduction services and did not define what constitutes “police presence” (e.g., marked vehicle, foot patrol, active enforcement, etc.). Future research should aim to more precisely characterize both the type and function of police presence and examine how different forms influence access to harm reduction sites. Finally, because our primary outcome relied on a six-month recall period, some study visits conducted shortly after January 31, 2023, may capture experiences that occurred both before and after the implementation of decriminalization. This raises the potential for non-differential exposure misclassification, as exposure status was assigned based on the date of the interview rather than the date the event occurred. Such misclassification would likely bias our results towards the null; therefore, potentially underestimating the true impact of decriminalization on policing-related barriers to harm reduction services. This limitation may be particularly relevant to the estimate for the “level change upon decriminalization”, as this variable assumes a discrete level change that begins precisely on January 31, 2023. In reality, changes to policing practices likely occurred gradually. Moreover, anticipatory behavior change—a well-documented phenomenon that occurs in response to awareness of the future implementation of a policy among affected groups—may have occurred as law enforcement adjusted practices in anticipation of decriminalization (Ahmad & Xiao, 2013; Alpert, 2016; Hyun & Milcheva, 2019; Llopis et al., 2023; Mueller, 2020). This is especially plausible in the study context, where decriminalization was highly publicized and preceded by other relevant policy changes, such as the August 2020 directive from the Public Prosecution Service to limit prosecutions for personal possession, effectively resulting in *de facto* decriminalization across Canada (Public Prosecution Service of Canada, 2020). Similar anticipatory trends were documented in Oregon, where data indicate that drug possession arrests declined following the passage—but prior to the enactment—of Measure 110 in February 2021, suggesting that police practices may have begun shifting in advance of formal legislative change (Davis et al., 2023).

In conclusion, the present study found that self-reported policing-related barriers to accessing harm reduction services among young people who use drugs, including young Indigenous people who use drugs, in Vancouver significantly declined following the implementation of decriminalization of personal possession. These findings suggest that decriminalization of personal possession may have advanced a central policy objective by reducing criminalization-related barriers to harm reduction services among people who use drugs. This evidence provides empirical support for the adoption of decriminalization of personal possession in other settings as a potential strategy to increase engagement with health services.

## Figures and Tables

**Table 1:** Baseline characteristics of study participants stratified by police barriers to accessing harm reduction sites, June 2021 to May 2024 (*n* = 319).

Characteristic	Total	Police barrier		*p*-value^†^
	
	*n* (%)	Yes	No	
	
	*n* = 319	*n* (%)	*n* (%)	
		*n* = 83 (26.0)	*n* = 236 (74.0)	

Age (median, Q1-Q3)	28.0 (24.9–30.4)	28.5 (25.2–32.1)	27.8 (24.9–29.8)	0.090
Gender				
Women	100 (31.3 %)	28 (33.7 %)	72 (30.5 %)	0.049
Transgender	6 (1.9 %)	0 (0.0 %)	6 (2.5 %)	
Two-Spirit	9 (2.8 %)	2 (2.4 %)	7 (3.0 %)	
Self-described	12 (3.8 %)	7 (8.4 %)	5 (2.1%)	
Man	192 (60.2 %)	46 (55.4 %)	146 (61.9 %)	
Race/ancestry (strongest identity)				
Indigenous	105 (32.9 %)	27 (32.5 %)	78 (33.5 %)	0.843
Black	13 (4.1 %)	4 (4.8 %)	9 (3.8 %)	
Person of Colour	31 (9.7 %)	6 (7.2 %)	25 (10.6 %)	
white	165 (51.7 %)	44 (53.0 %)	121 (51.3 %)	
Indigenous ancestry				
Yes	138 (43.3 %)	39 (47.0 %)	99 (41.9 %)	0.436
No	176 (55.2 %)	42 (50.6 %)	134 (56.8 %)	
Born in Canada				
Yes	295 (92.5 %)	79 (95.2 %)	216 (91.5 %)	0.214
No	22 (6.9 %)	3 (3.6 %)	19 (8.1 %)	
Self-identified 2SLGBT identity				
Yes	113 (35.4 %)	31 (37.3 %)	82 (34.7 %)	0.690
No	206 (64.6 %)	52 (62.7 %)	154 (65.3 %)	
Education				
≥ high school	162 (50.8 %)	40 (48.2 %)	122 (51.7 %)	0.610
< high school	154 (48.3 %)	42 (50.6 %)	112 (47.5 %)	
Experiencing homelessness*				
Yes	131 (41.1 %)	38 (45.8 %)	93 (39.4 %)	0.364
No	187 (58.6 %)	45 (54.2 %)	142 (60.2 %)	
Current unstable housing				
Yes	211 (66.1 %)	63 (75.9 %)	148 (62.7 %)	0.042
No	107 (33.5 %)	20 (24.1 %)	87 (36.9 %)	
DTES residence*				
Yes	77 (24.1 %)	19 (22.9 %)	58 (24.6 %)	0.769
No	242 (75.9 %)	64 (77.1 %)	178 (75.4 %)	
Regular employment*				
Yes	130 (40.8 %)	31 (37.3 %)	99 (41.9 %)	0.517
No	189 (59.2 %)	52 (62.7 %)	137 (58.1 %)	
Sex work*				
Yes	29 (9.1 %)	13 (15.7 %)	16 (6.8 %)	0.014
No	284 (89.0 %)	66 (79.5 %)	218 (92.4 %)	
Drug dealing*				
Yes	135 (42.3 %)	49 (59.0 %)	86 (36.4 %)	*<*0.001
No	182 (57.1 %)	33 (39.8 %)	149 (63.1 %)	
Illegal income generation*				
Yes	77 (24.1 %)	29 (34.9 %)	48 (20.3 %)	0.011
No	242 (75.9 %)	54 (65.1 %)	188 (79.7 %)	
Incarcerated*				
Yes	42 (13.2 %)	13 (15.7 %)	29 (12.3 %)	0.444
No	269 (84.3 %)	65 (78.3 %)	204 (86.4 %)	
Non-fatal overdose*				
Yes	66 (20.7 %)	20 (24.1 %)	46 (19.5 %)	0.338
No	247 (77.4 %)	59 (71.1 %)	188 (79.7 %)	
Any injection drug use*				
Yes	128 (40.1 %)	41 (49.4 %)	87 (36.9 %)	0.050
No	189 (59.2 %)	41 (49.4 %)	148 (62.7 %)	
≥ Weekly unregulated opioid* ^a^				
Yes	172 (53.9 %)	59 (71.1 %)	113 (47.9 %)	<0.001
No	147 (46.1 %)	24 (28.9 %)	123 (52.1 %)	
≥ Weekly cocaine*				
Yes	21 (6.6 %)	3 (3.6 %)	18 (7.6 %)	0.303
No	295 (92.5 %)	78 (94.0 %)	217 (91.9 %)	
≥ Weekly crack*				
Yes	36 (11.3 %)	9 (10.8 %)	27 (11.4 %)	1.000
No	280 (87.8 %)	72 (86.7 %)	208 (88.1 %)	
≥ Weekly crystal methamphetamine*				
Yes	170 (53.3 %)	56 (67.5 %)	114 (48.3 %)	0.003
No	149 (46.7 %)	27 (32.5 %)	122 (51.7 %)	
Binge drug use*				
Yes	29 (9.1 %)	5 (6.0 %)	24 (10.2 %)	1.000
No	15 (4.7 %)	3 (3.6 %)	12 (5.1 %)	
Inject drugs alone*				
Yes	85 (26.6 %)	26 (31.3 %)	59 (25.0 %)	0.247
No	230 (72.1 %)	55 (66.3 %)	175 (74.2 %)	
Public injection*				
Yes	79 (24.8 %)	28 (33.7 %)	51 (21.6 %)	0.038
No	236 (74.0 %)	54 (65.1 %)	182 (77.1 %)	
Opioid agonist therapy*				
Yes	111 (34.8 %)	36 (43.4 %)	75 (31.8 %)	0.044
No	201 (63.0 %)	44 (53.0 %)	157 (66.5 %)	
Drug/alcohol treatment*				
Yes	50 (15.7 %)	11 (13.3 %)	39 (16.5 %)	0.598
No	258 (80.9 %)	68 (81.9 %)	190 (80.5 %)	
Barriers to accessing addiction treatment*				
Yes	38 (11.9 %)	13 (15.7 %)	25 (10.6 %)	0.231
No	270 (84.6 %)	65 (78.3 %)	205 (86.9 %)	
Moderate or severe depression^b^				
Yes	109 (34.2 %)	29 (34.9 %)	80 (33.9 %)	0.459
No	145 (45.5 %)	32 (38.6 %)	113 (47.9 %)	
Moderate or severe anxiety^b^				
Yes	149 (46.7 %)	43 (51.8 %)	106 (44.9 %)	0.040
No	108 (33.9 %)	19 (22.9 %)	89 (37.7 %)	
Number of study visits (median, standard deviation [SD]; Q1-Q3)	3.0 (1.8); (1.0–1.0)	4.0 (1.7); (2.0–5.0)	2.0 (1.7); (1.0–4.0)	<0.001

Q1-Q3 = first to third quartile.

Race and ancestry categories are not mutually exclusive.

All column percentages may not sum to 100 % due to missing data.

* Denotes activities in the last 6 months.

† Tested via exact chi-square test (binary) or Kruskal-Wallis test (continuous).

a Unregulated opioids includes fentanyl, down unspecified, heroin, or other illicit opioids.

b Refers to the 7 days prior to the interview.

**Table 2:** Unadjusted and adjusted multivariable generalized estimating equations (GEE) models comparing levels and trends of police barriers to harm reduction services before, upon and after the decriminalization of personal possession among young people who use drugs, June 2021-May 2024 (*n* = 319).

Parameter	Unadjusted Odds Ratio (95 % CI); *p*-value	Adjusted Odds Ratio* (95 % CI); *p*-value

Trend before decriminalization	2.22 (1.41,3.50); 0.001	2.41 (1.29,4.51); 0.006
Level change upon decriminalization	0.61 (0.32,1.17); 0.137	0.35 (0.15,0.82); 0.015
Trend after decriminalization ^†^	0.57 (0.27,1.20); 0.142	1.12 (0.48,2.58); 0.794

* Multivariable models adjusted for the following variables: homelessness; unstable housing; barriers to addiction treatment; ≥ weekly crystal methamphetamine use; drug dealing; moderate/severe anxiety; public injection

† Computed by reparametrizing the model

**Table 3:** Baseline characteristics of Indigenous participants stratified by experiencing police barriers to accessing harm reduction sites, June 2021 to May 2024 (*n* = 138).

Characteristic	Total	Police barrier		*p*-value^†^
	
	*n* (%)	Yes	No	
	*n* = 138	*n* (%)	*n* (%)	
		*n* = 39 (28.3)	*n* = 99 (71.7)	

Age (median, Q1-Q3)	27.1 (24.8–29.8)	28.5 (24.9–30.4)	26.9 (24.6–29.8)	0.280
Gender				
Women	44 (31.9 %)	12 (30.8 %)	32 (32.3 %)	0.108
Transgender	1 (0.7 %)	0 (0.0 %)	1 (1.0 %)	
Two-Spirit	8 (5.8 %)	2 (5.1 %)	6 (6.1 %)	
Self-described	5 (3.6 %)	4 (10.3 %)	1 (1.0 %)	
Man	80 (58.0 %)	21 (53.8 %)	59 (59.6 %)	
Race/ancestry (strongest identity)				
Indigenous	105 (76.1 %)	27 (69.2 %)	78 (78.8 %)	0.265
Black	2 (1.4 %)	0 (0.0 %)	2 (2.0 %)	
Person of Colour	5 (3.6 %)	1 (2.6 %)	4 (4.0 %)	
white	26 (18.8 %)	11 (28.2 %)	15 (15.2 %)	
Self-identified 2SLGBT identity				
Yes	56 (40.6 %)	19 (48.7 %)	37 (37.4 %)	0.251
No	82 (59.4 %)	20 (51.3 %)	62 (62.6 %)	
Education				
≥ high school	71 (51.4 %)	20 (51.3 %)	51 (51.5 %)	1.000
≤ high school	67 (48.6 %)	19 (48.7 %)	48 (48.5 %)	
Experiencing homelessness*				
Yes	60 (43.5 %)	16 (41.0 %)	44 (44.4 %)	0.707
No	77 (55.8 %)	23 (59.0 %)	54 (54.5 %)	
Current unstable housing				
Yes	99 (71.7 %)	28 (71.8 %)	71 (71.7 %)	1.000
No	39 (28.3 %)	11 (28.2 %)	28 (28.3 %)	
DTES residence*				
Yes	34 (24.6 %)	5 (12.8 %)	29 (29.3 %)	0.050
No	104 (75.4 %)	34 (87.2 %)	70 (70.7 %)	
Regular employment*				
Yes	53 (38.4 %)	18 (46.2 %)	35 (35.4 %)	0.250
No	85 (61.6 %)	21 (53.8 %)	64 (64.6 %)	
Sex work*				
Yes	15 (10.9 %)	7 (17.9 %)	8 (8.1 %)	0.126
No	120 (87.0 %)	31 (79.5 %)	89 (89.9 %)	
Drug dealing*				
Yes	61 (44.2 %)	24 (61.5 %)	37 (37.4 %)	0.014
No	76 (55.1 %)	15 (38.5 %)	61 (61.6 %)	
Illegal income generation*				
Yes	35 (25.4 %)	12 (30.8 %)	23 (23.2 %)	0.389
No	103 (74.6 %)	27 (69.2 %)	76 (76.8 %)	
Incarcerated*				
Yes	19 (13.8 %)	6 (15.4 %)	13 (13.1 %)	0.786
No	115 (83.3 %)	32 (82.1 %)	83 (83.8 %)	
Non-fatal overdose*				
Yes	24 (17.4 %)	7 (17.9 %)	17 (17.2 %)	0.802
No	110 (79.7 %)	29 (74.4 %)	81 (81.8 %)	
Any injection drug use*				
Yes	50 (36.2 %)	17 (43.6 %)	33 (33.3 %)	0.327
No	87 (63.0 %)	22 (56.4 %)	65 (65.7 %)	
≥ Weekly unregulated opioid*^a^				
Yes	68 (49.3 %)	25 (64.1 %)	43 (43.4 %)	0.037
No	70 (50.7 %)	14 (35.9 %)	56 (56.6 %)	
≥ Weekly cocaine*				
Yes	9 (6.5 %)	1 (2.6 %)	8 (8.1 %)	0.289
No	128 (92.8 %)	38 (97.4 %)	90 (90.9 %)	
≥ Weekly crack*				
Yes	20 (14.5 %)	3 (7.7 %)	17 (17.2 %)	0.186
No	117 (84.8 %)	36 (92.3 %)	81 (81.8 %)	
≥ Weekly crystal methamphetamine*				
Yes	76 (55.1 %)	26 (66.7 %)	50 (50.5 %)	0.092
No	62 (44.9 %)	13 (33.3 %)	49 (49.5 %)	
Binge drug use*				
Yes	18 (13.0 %)	2 (5.1 %)	16 (16.2 %)	1.000
No	7 (5.1 %)	1 (2.6 %)	6 (6.1 %)	
Inject drugs alone*				
Yes	33 (23.9 %)	13 (33.3 %)	20 (20.2 %)	0.125
No	104 (75.4 %)	26 (66.7 %)	78 (78.8 %)	
Public injection*				
Yes	32 (23.2 %)	13 (33.3 %)	19 (19.2 %)	0.116
No	105 (76.1 %)	26 (66.7 %)	79 (79.8 %)	
Opioid agonist therapy*				
Yes	45 (32.6 %)	18 (46.2 %)	27 (27.3 %)	0.043
No	89 (64.5 %)	20 (51.3 %)	69 (69.7 %)	
Drug/alcohol treatment*				
Yes	17 (12.3 %)	3 (7.7 %)	14 (14.1 %)	0.393
No	116 (84.1 %)	35 (89.7 %)	81 (81.8 %)	
Barriers to accessing addiction treatment*				
Yes	17 (12.3 %)	4 (10.3 %)	13 (13.1 %)	0.778
No	117 (84.8 %)	34 (87.2 %)	83 (83.8 %)	
Moderate or severe depression^b^				
Yes	40 (29.0 %)	9 (23.1 %)	31 (31.3 %)	0.817
No	65 (47.1 %)	17 (43.6 %)	48 (48.5 %)	
Moderate or severe anxiety^b^				
Yes	64 (46.4 %)	17 (43.6 %)	47 (47.5 %)	0.821
No	43 (31.2 %)	10 (25.6 %)	33 (33.3 %)	
Number of study visits (median, standard deviation [SD]; Q1-Q3)	2.0 (1.7); (1.0–4.0)	4.0 (1.7); (2.0–5.0)	2.0 (1.5); (1.0–3.0)	<0.001

Q1-Q3 = first to third quartile.

Race and ancestry categories are not mutually exclusive.

All column percentages may not sum to 100 % due to missing data.

* Denotes activities in the last 6 months.

† Tested via exact chi-square test (binary) or Kruskal-Wallis test (continuous).

a Unregulated opioids includes fentanyl, down unspecified, heroin, or other illicit opioids.

b Refers to the 7 days prior to the interview.

**Table 4:** Unadjusted and adjusted multivariable generalized estimating equations (GEE) models comparing levels and trends of experiencing police-related barriers to harm reduction services before, upon and after decriminalization of personal possession among young Indigenous people who use drugs, June 2021-May 2024 (*n* = 138).

Parameter	Unadjusted Odds Ratio (95 % CI); *p*-value	Adjusted Odds Ratio* (95 % CI); *p*-value

Trend before decriminalization	2.58 (1.23,5.41); 0.012	2.44 (1.11,5.37); 0.027
Level change upon decriminalization	0.70 (0.25,1.94); 0.487	0.66 (0.22,1.98); 0.456
Trend after decriminalization ^†^	0.24 (0.08,0.76); 0.015	0.28 (0.08,0.97); 0.045

* Multivariable models adjusted for the following variables: ≥ weekly crack use; ≥ weekly crystal methamphetamine use; and opioid agonist therapy.

† Computed by reparametrizing the model
